# Solid-phase synthesis of imprinted nanoparticles as artificial antibodies against the C-terminus of the cannabinoid CB1 receptor: exploring a viable alternative for bioanalysis

**DOI:** 10.1007/s00604-021-05029-z

**Published:** 2021-10-07

**Authors:** Alberto Gómez-Caballero, Ainhoa Elejaga-Jimeno, Gontzal García del Caño, Nora Unceta, Antonio Guerreiro, Miquel Saumell-Esnaola, Joan Sallés, M. Aránzazu Goicolea, Ramón J. Barrio

**Affiliations:** 1 Department of Analytical Chemistry, University of the Basque Country UPV/EHU, 01006 Vitoria-Gasteiz (Álava), Spain; 2 Department of Neurosciences, Faculty of Pharmacy, University of the Basque Country UPV/EHU, 01006 Vitoria-Gasteiz (Álava), Spain; 3 MIP Diagnostics, Leicester, UK; 4 Department of Pharmacology, Faculty of Pharmacy, University of the Basque Country UPV/EHU, 01006 Vitoria-Gasteiz (Álava), Spain; 5 Centro de Investigación Biomédica en Red de Salud Mental (CIBERSAM), 28029 Madrid, Spain

**Keywords:** Artificial antibody, Epitope imprinting, GPCR, CB1 receptor, Molecularly imprinted nanoparticles, Bioanalysis

## Abstract

The production of artificial anti-CB1 antibodies in nanoparticle format is described using the solid-phase imprinting approach. Instead of whole protein imprinting, a linear C-terminus sequence of the receptor comprising 15 amino acids (458-KVTMSVSTDTSAEAL-472) has been used as template, in accordance with the epitope imprinting approach. This sequence is located intracellularly, and it is involved in coupling to G_i/o_ proteins, being responsible for CB1 receptor desensitisation and internalisation. Developed molecularly imprinted materials were found to be in the nanometre scale, with a particle size of 126.4 ± 10.5 nm at pH 3 (25 ºC) and spherical shape. It was also observed that the size was sensible to temperature changes being reduced to 106.3 ± 15.2 nm at 35 °C. Lower critical solution temperature of this polymer was found to be ≈ 33.4 °C. The affinity and selectivity of the artificial antibody were assessed through dot blot and Western blot experiments. For the latter, recombinant fusion proteins GST-CB1_414-472_ and GST-CB1_414-442_ were produced to work respectively as target and negative control proteins. The control protein did not carry the target epitope for being devoid of last 30 amino acids at the C-terminus. The results demonstrated that the anti-CB1 material recognised selectively the target protein, thanks to the presence of the 15-amino acid sequence selected as epitope, which revealed that binding occurred at the C-terminus of the receptor itself. The methodology presented may pave the way for the development of novel imprinted nanomaterials for other proteins included in the superfamily of the G-protein-coupled receptors (GPCR).

**Graphical abstract:**

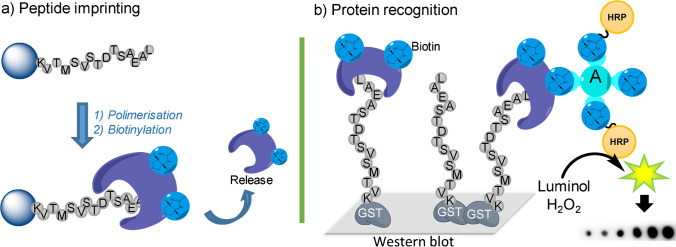

**Supplementary Information:**

The online version contains supplementary material available at 10.1007/s00604-021-05029-z.

## Introduction

Developing artificial materials having antibody-like molecular recognition ability has experienced a fast expansion over the last decade. For this purpose, researchers have emphasised on imprinting macromolecules on synthetic polymers as a way to yield artificial antibody analogues [1]. Molecularly imprinted polymers (MIP), sometimes referred to as plastic antibodies, are materials most often developed by copolymerisation of vinyl and/or acrylic type of functional and cross-linking monomers in the presence of a template [2]. Traditionally, these materials were synthesised by free radical polymerisation (FRP) in bulk format [3]. However, bulk MIPs usually present a high number of non-specific sites, which contribute to cross-reactivity and thus limit their application in clinical diagnostics, drug delivery, chemical sensing, or separation techniques [4]. As an effort to overcome the aforementioned drawbacks, controlled or living radical polymerisation (CRP) strategies have been combined with imprinting technology achieving improved recognition properties. CRP allows for the control over polymer architecture, providing an improved inner morphology due to a more homogeneous distribution of cross-linking points [5]. Hence, obtained MIPs resemble more to monoclonal antibodies, showing (pseudo)monoclonal binding behaviour with less cross-reactivity [3].

Many of these challenges have also been addressed, thanks to the relatively recent emergence of nano-sized MIPs [6], which have been fabricated using different strategies, including the recently emerged solid-phase synthesis [7–10]. Unlike other strategies, it relies on having the template covalently immobilised on a solid support, instead of having it free in solution. Immobilised templates present reduced number of degrees of freedom to establish monomer-template complexes, thereby increasing the quality of the imprinting event [11]. This strategy may be adapted for oriented coupling of peptides or proteins, making all binding sites have the same orientation [12]. Accordingly, created binding sites will hence recognise the same amino acid sequence (epitope) of the template, and this will also contribute to MIP monoclonality [13].

The versatility of solid-phase synthesis has been empirically demonstrated both for templates having low molecular weight [7, 14] and macromolecules (> 1500 Da) [12, 15, 16]. However, for macromolecular templates, especially proteins, imprinting is still a challenge due to size restrictions, the poor stability at non-physiological environments [15], and the large number of functionalities, which may contribute to the creation of multiple types of binding sites with different affinities and specificities [4]. In order to circumvent these problems, different scientists considered imprinting a small exposed fragment of a larger molecule instead of imprinting the whole protein itself [17, 18]. The proposed strategy was called the epitope approach, and it imitates the binding behaviour of natural antibodies, which recognise a certain sequence of their target, namely, an epitope. Epitope imprinting has attracted much attention in bioanalysis, therapy, and imaging as an alternative to natural antibodies [19, 20]. Most often, linear peptide fragments that consist of 6–12 amino acids are the predominant ones for imprinting [4, 21, 22]; however, longer peptides [23] or even nonlinear conformational epitopes, which are present in folded proteins or fragments, have also been employed [19].

Considering potential benefits provided by epitope imprinting at nanoscale level, molecularly imprinted nanoparticles (MIN) as anti-CB1 artificial antibodies have been produced here, which bind specifically to the C-terminus of the CB1 receptor of the endocannabinoid system. The presence of the CB1 receptor in the central nervous system (CNS) [24] explains many pharmacological effects associated to delta-9-tetrahydrocannabinol (THC), the main active substance of the plant *Cannabis sativa*, and the pathological regulation of its expression has been involved in a series of psychiatric (addiction, depression and anxiety) [25] and neurologic disorders (epilepsy, multiple sclerosis, and neuropathic pain) [26]. All these are very relevant due to their high prevalence and the lack of efficient pharmacological treatments. CB1 receptor presents a structure defined by an array of seven membrane-spanning helices with intervening intracellular loops and a C-terminal domain that can associate with G proteins [27]. The intracellular region is crucial to determine receptor functionality with regard to its coupling with G_i/o_ proteins and its activation, receptor desensitisation, and internalisation [28]. Accordingly, an epitope that corresponds to the C-terminal tail of the receptor positioned intracellularly has been selected as target for this work, having a sequence of 15 amino acids (458-KVTMSVSTDTSAEAL-472) located outside the α-H9 helix. This epitope will serve as template for the synthesis of MIPs at nanoscale level that will be exploited as antibody substitutes for the study of the CB1 receptor, which will allow for the determination of its expression and regulation in a wide variety of disorders. Especially considering the limited specificity and reproducibility presented so far by natural antibodies employed in this context [29].

## Experimental

### Materials and apparatus

**Fig. 1:**
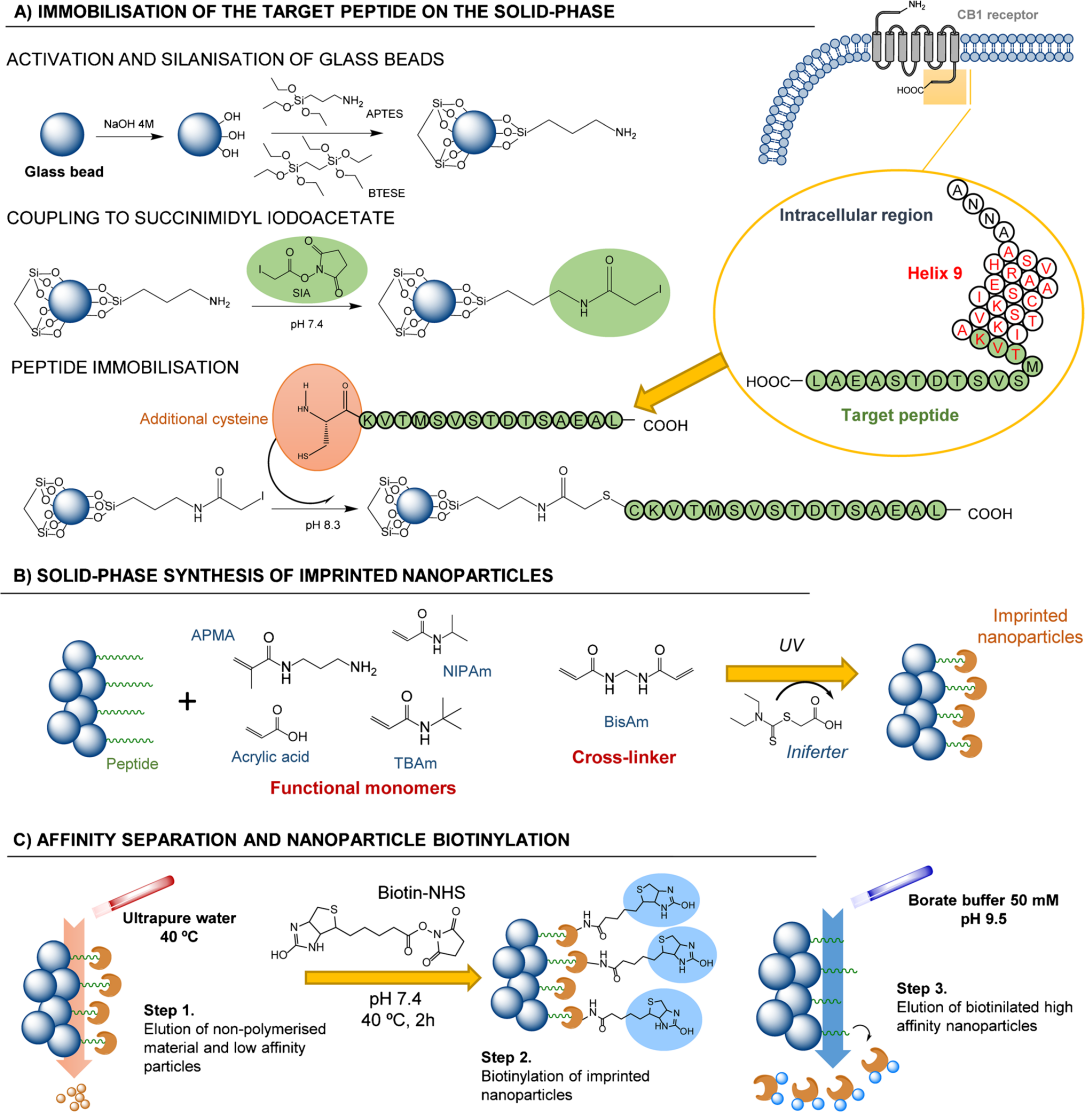
Schematic representation of the entire protocol employed to synthesise biotinylated molecularly imprinted nanoparticles

Target peptide (purity 96.76%, Maldi-TOF (*m/z*): (M + H^+^) 1643.57) containing an additional cysteine residue (C-KVTMSVSTDTSAEAL) (Fig. 1) was custom-synthesised by Caslo ApS (Denmark). As control peptide (purity 98.38%, Maldi-TOF (*m/z*): (M + H^+^) 1717.26) a 15-amino acid sequence also containing an additional cysteine was used (C-HRAAESCIKSTVKIA). This sequence can be found at the intracellular helix 9 motif of the CB1 receptor [28]. Both peptides were analysed using the software Agadir (http://agadir.crg.es/) in order to estimate their propensity to create α-helixes in aqueous solutions. Accordingly, it was observed that, at pH, temperature and ionic strength values used at this work, theoretical peptide tendency to create α-helixes was always below 1%, which denotes that selected epitopes will be in linear state at all working conditions.

For peptide immobilisation, glass beads with a diameter of 150–212 µm were acquired from Merck (Madrid, Spain). Succinimidyl iodoacetate (SIA) (> 97%) was purchased from Thermo Fisher Scientific (Spain), and cysteamine hydrochloride (> 98%) was acquired from Merck (Spain).

The silanes 3-aminopropyltriethoxysilane (APTES) (99%) and 1,2-bis(triethoxysilyl)ethane (BTESE) (95%), and the monomers N-isopropylacrylamide (NIPAm) (97%), N-tert-butylacrylamide (TBAm) (97%), acrylic acid (AA) (99%), N-(3-aminopropyl) methacrylamide hydrochloride (APMA) (98%), and N,N’-methylenebis(acrylamide) (BisAm) (99%) were obtained from Merck (Spain). The water-soluble photoiniferter diethylthiocarbamoylsulfanyl acetic acid (DCAA) was synthesised as described elsewhere [30] using sodium diethyldithiocarbamate (98%) and sodium chloroacetate (98%) acquired from Alfa-Aesar (Spain) (yield 51.3%). High-resolution mass spectra for the iniferter having a chemical formula C_7_H_13_NO_2_S_2_ were obtained by positive-ion electrospray ionisation (ESI-QTOF-MS). *m/z*, (M + H) + 208.0464; calculated mass, 207.0388; found mass, 207.0388; difference, 0.34 ppm.

Synthesised nanoparticles were biotinylated using ( +)-biotin N-hydroxysuccinimide ester (biotin-NHS) (98%) purchased from Merck (Spain).

Fatty acid free bovine serum albumin (BSA) (99%), used for blotting experiments, was purchased from Merck (Spain) while Tween-20 was acquired from Alfa Aesar (Spain).

HPLC-grade ethanol (EtOH) and acetonitrile (ACN) and reagent-grade acetone and methanol were purchased from Scharlab (Spain), whereas dimethyl sulfoxide was acquired from Panreac (Spain). Buffer solutions were prepared with ultrapure water (resistivity of 18.2 MΩ.cm), which was obtained using Elix20 reverse osmosis and Milli-Q water purification systems from Merck (Spain). All other reagents were analytical grade and they were used as received.

The *Apparatus* section has been moved to Supplementary information.

### Immobilisation of the target peptide on the solid-phase

Figure 1 depicts schematically the immobilisation protocol for the target peptide. First, 120 g of glass beads (GB) were activated in a boiling solution of NaOH 4 M for 30 min. Next, the beads were washed with water and methanol and dried in an oven at 100 °C. Activated GB were immersed in a 100 mL 95:5 ethanol:water solution acidified with 1 mL acetic acid and then heated up to 70 °C. Immediately after, 3 mL APTES and 0.5 mL BTESE were added (10:1 APTES:BTESE molar ratio) and it was left to react overnight at RT. Thereafter, silanised GB were filtered, washed with methanol and acetone, and then dried in a desiccator under vacuum. Finally, for complete water removal, they were placed in an oven at 150 °C for 1 h. To determine the grafting degree of APTES, the ninhydrin test was performed as detailed by the manufacturer; to this end, 1 mg of silanised GB and 2% ninhydrin solution were used from Merck (Spain), which resulted in a grafting degree of 544 ± 66 µg APTES per g of GB.

Thirty grams of dry GB bearing NH_2_ groups were incubated for 2 h in 25 mL of a 1 mg/mL solution of SIA, prepared in 0.1 M phosphate buffered saline containing 0.1 M NaCl at pH 7.4 (PBS). Then, the beads were filtered, washed thoroughly with water and acetonitrile, and immersed in a 25 mL solution of 10 mM sodium borate and 5 mM EDTA (pH 8.3), which contained 10 mg of the target peptide. After overnight reaction at 4 °C, the GB were filtered, washed repeatedly with 0.1 M PBS and water, and immersed in 25 mM phosphate buffer at pH 7.4 (PB) to proceed with the solid-phase synthesis. The amount of peptide bound to the solid support was estimated using the Pierce BCA protein assay kit (Fisher Scientific, Spain), using a sample of 1 g of peptide-bound GB. As a result 51.5 ± 7.3 µg of bound peptide per g of GB was observed.

### Solid-phase synthesis of imprinted nanoparticles

For molecularly imprinted nanoparticle (MIN) synthesis, 72.7 mg of NIPAm (0.623 mmol, 43.75% over total moles of functional monomers), 70 mg of TBAm (0.534 mmol; 37.5%), 32.1 mg of APMA (0.178 mmol; 12.5%), 6 µL of AA (0.089 mmol; 6.25%), and 11.5 mg of BisAm (0.075 mmol) as cross-linker were added to 25 mL of PB, containing 30 mg of peptide-bound GB. A total monomer amount of 1.5 mmol was used, 5% of which were relative to the cross-linker. This mixture was adapted from [31] and [32] taking into consideration the percentage of acidic, basic, hydrophobic, and hydrophilic amino acids present in the target peptide. To avoid monomer loss due to volatilisation, the solvent (PB buffer) was initially purged with N_2_ for 30 min in a round bottom glass tube sealed with a rubber septum. Then, all monomers were added and the mixture was degassed for another 5 min while being in an ice bath. After this, 11 mg of the water-soluble iniferter (DCAA) were added (3.5% of total monomer moles) and the tube was placed horizontally between two UV lamps to proceed with the polymerisation, irradiating the mixture from the top and the bottom for 30 min. Within the first 15 min, the mixture was placed in an ice bath during irradiation, whereas the last 15 min was conducted at RT.

After polymerisation, the GB were transferred to a 60-mL solid-phase extraction cartridge fitted with a polyethylene frit (20-µm porosity) from Merck (Spain) to perform a temperature-based affinity separation of synthesised MIN [9]. Initially, a warm washing step was carried out to remove low-affinity MIN, small oligomers, and unreacted compounds. To this end, the cartridge was preconditioned at 40 °C using a water bath and, thereafter, 10 × 20 mL of ultrapure water at 40 °C was percolated through it using a Vac Elut vacuum manifold (Agilent Technologies, Spain). Twenty millilitres corresponds approximately to the bed volume. After this step, high-affinity MIN will stay bound to the solid phase, whereas reagents used for polymerisation will be removed. At this point, biotinylation of MIN was performed in order for the particles to be usable in subsequent Western blot and dot blot experiments. To this end, 5 mg of biotin-NHS was dissolved in 1 mL of DMSO and 100 µL of this solution was added to 25 mL PB solution. This mixture was immediately added to the SPE cartridge containing the MIN bound to the GB, and it was left to react for 2 h in the dark. Afterwards, unreacted biotin was removed percolating 5 × 20 mL of ultrapure water through the cartridge at RT.

Elution of biotinylated MIN from the solid support was performed through cold washings at 6 °C. To this end, the cartridge was preconditioned in an ice bath and, thereafter, 8 × 20 mL of borate buffer 50 mM at 6 °C adjusted to pH 9.5 was percolated, reaching a final volume of MIN suspension of 160 mL. This was concentrated down using a rotatory evaporator obtaining a concentrated solution of about 30 mL of MIN, which was extensively dialysed through 6000–8000 KDa membranes (Spectrum Chemical, USA) in ultrapure water to remove salts. Then, it was further concentrated using an Amicon Ultra-15 centrifugal filter and washed repeatedly with ultrapure water, which served for further removing any remaining unreacted compound or salt. Finally, the nanoMIP suspension (around 2 mL) was transferred to a tube and water was evaporated to dryness using a sample concentrator DB200/3 from Techne (UK). The solid residue was weighed using a high-resolution ME215 balance from Sartorius (Spain) with a precision of 0.01 mg. Each synthesis produced 10.25 ± 0.81 mg of polymer (0.34 ± 0.03 mg.g^−1^ of GB) which is in accordance with other works [31, 32]. After weigh determination, solid MIN were resuspended in 10 mL of formate buffer 25 mM at pH 4 to maintain colloidal stability.

### Column packing and HPLC experiments

Different stationary phases were prepared for liquid chromatography experiments. The main one, used as target, consisted of GB having the target peptide grafted on its surface (the grafting protocol is detailed in the “Experimental” section), while the others, used as control or blank stationary phases, were respectively made of the control peptide or cysteamine grafted to the beads. Cysteamine was used to couple to pendant SIA units giving rise to a blank column having no peptide attached. The used protocol to attach either the control peptide or cysteamine onto functionalised GB was the same as the one used for the target peptide.

Three grams of GB having the target peptide, control peptide, or cysteamine immobilised on its surface were individually packed in stainless columns of 150 mm in length and 4.6 mm in diameter. The packing was performed using the Pack in a Box column packing system from Restek (USA) comprised of a dual piston pump and a 20-mL reservoir using water as the pushing solvent. Column packing was started at 1 mL min^−1^ and gradually increased to 20 mL min^−1^ until constant pressure was observed for, at least, 10 min. Then, it was reduced to 1 mL min^−1^ for another 5 min. HPLC experiments were carried at a flow rate of 0.4 mL min^−1^, using a liquid chromatograph coupled to a fluorescence detector working at 242 nm and 518 nm as excitation and emission wavelengths, respectively. Injection volume was 20 µL for all experiments. Column thermostat was set to the desired temperature and it was left to stabilise for 30 min prior to nanoparticle injections.

### Dot blot and Western blot experiments

MIN-peptide binding was assessed by dot blot experiments using nitrocellulose membranes. Spots of 1 µL having known concentrations of the target and control peptides were blotted on the membrane and it was left to dry. Thereafter, non-specific sites were blocked using 0.5% BSA in 0.1 M PBS (pH 7.4) for 1 h at RT; afterwards, the membranes were incubated in a 1:5000 diluted stock suspension of 510 mg mL^−1^ of MIN at 35 ºC in 0.1 M PBS for 2.5 h. Finally, they were washed for 10 min in 0.1 M PBS containing 0.2% Tween 20 (PBS-T) three times. All these steps were performed under constant circular shaking.

Binding of biotinylated MIN to the peptide on nitrocellulose membranes was quantified by chemiluminescence. For this purpose, the commercial Vectastain ABC-HRP Kit was used (Vector laboratories, UK), which contains avidin (Reagent A) and biotinylated horseradish peroxidase (HRP) (Reagent B). Ten microliters of reagent A of the kit and another 10 µL of reagent B were added to 10 mL of PBS-T and it was left to react for 1 h. Then, the nitrocellulose membranes were immersed into the mixture and they were left to react with the avidin–biotin/HRP complex for 1 h. Thereafter, they were washed three times with PBS-T and then with PBS. Finally, for image acquisition, an Image Quant 350 imager was used, which was acquired from GE Life Sciences (Spain). Dots were visualized using the Clarity Western ECL Substrate from Bio-Rad (Spain) according to the manufacturer’s recommendations.

For Western blot experiments recombinant fusion proteins, GST-CB1_414-472_ and GST-CB1_414-442_, were produced as described in supporting information. An equimolar mixture of 3.6 pmol µL^−1^ of each protein was prepared as stock solution, and it was further diluted using urea 4 × buffer (supplementary information). Achieved final concentrations of recombinant proteins were 0.075, 0.15, 0.30, 0.60, 1.2, 1.8, and 3.6 pmol µL^−1^. Once they were denaturalised, 10 µL of each dilution was loaded on the gel and electrophoresis was run at 120 V in a Protean II xi cell from Bio-Rad (Spain). After migration, proteins were transferred to Immun-Blot PVDF membranes using a Trans-Blot transfer system (Bio-Rad, Spain) applying 30 V for 18 h. Thereafter, the membranes were gently washed with ultrapure water and immersed in an aqueous solution containing 7% acetic acid and 10% methanol for protein fixation. The membranes were incubated in this solution for 15 min at RT under gentle shaking and, afterwards, they were washed with ultrapure water for 10 min at RT, and another two times using PBS-T. At this point, the membranes were ready for being blocked and incubated with the MIN suspension as detailed above for dot blot experiments.

## Results and discussion

### Assessment of the solid-phase synthesis protocol using liquid chromatography

Liquid chromatography was considered as a tool to determine the best conditions at which MIN-peptide binding occurred on the surface of GB. It was hypothesised that, after peptide attachment to GB (Experimental section), these beads could be packed inside an HPLC column and use them as stationary phase. In this context, if a suspension of MIN passes across the column, part of those nanoparticles will be trapped due to peptide-MIN interactions, while non-bound particles will elute and quantified in the fluorescence detector. For that purpose, synthesised MIN were provided with fluorescence using N-fluoresceinyl acrylamide (supplementary information). All these chromatographic experiments were used to assess MIN binding to the target peptide and were found to be helpful to determine the best conditions to carry out solid-phase synthesis and subsequent affinity-based washes.

**Fig. 2:**
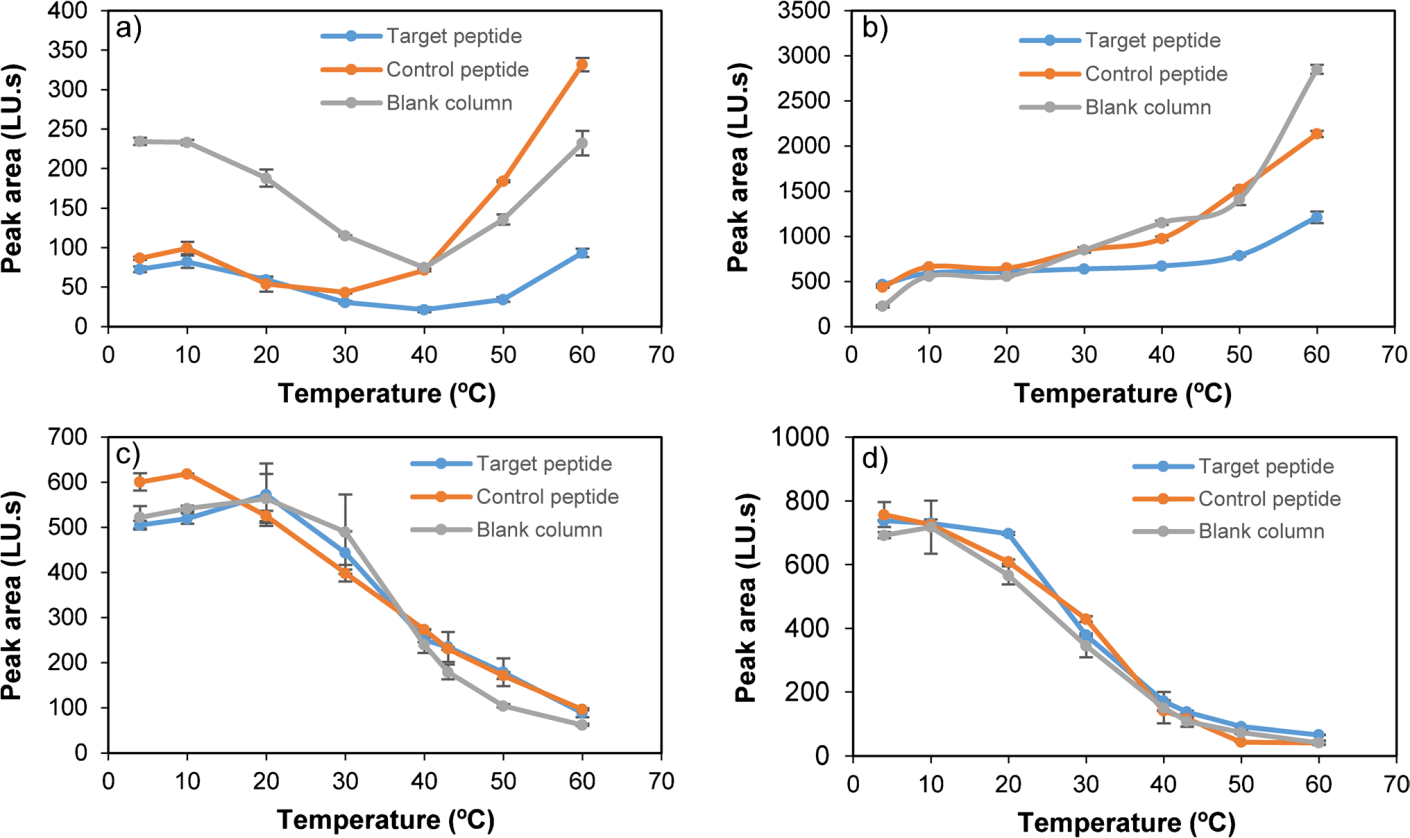
Peak areas relative to unbound fluorescent MIN using **a** water, **b** ethanol, **c** 25 mM PB (pH:7.4), and **d** 0.1 M PBS (pH:7.4) as mobile phases

First, temperature effect on MIN binding was studied. For this purpose, target peptide, control peptide, and the blank column (containing cysteamine) (supplementary information) were preheated at different temperatures. Then, the suspension of fluorescent MIN nanoparticles was injected, and unbound MIN quantified, which passed through the column and gave rise to a chromatographic peak. Figure 2a depicts peak area values corresponding to unbound MIN at different temperatures. As it can be observed, minimum peak area is registered at 30–40 °C for all stationary phases, which means stronger binding, that is, less elution. At lower or higher temperatures, MIN elution increases, especially with stationary phases containing the control peptide or cysteamine. Below 30 °C, MIN binds almost equally to the target peptide or the control one; this might suggest that, at this point, retention is manly governed by non-specific interactions. In contrast, above 30 °C, retention at the column containing the target peptide is significantly higher, which demonstrates that created binding sites are responsible for MIN-peptide binding. These experiments clearly demonstrate temperature-based behaviour of created MIN, which is definitely in agreement with other works, suggesting that the imprinted material presents thermoresponsive character [17]. In this context, temperatures below the lower critical solution temperature (LCST) contribute to particle swelling, as they turn more hydrophilic. Consequently, the size of binding sites increases and, therefore, a selectivity loss may occur. This may explain the reason why both the target and control peptides show similar binding below 30 °C. Comparatively, the blank column shows poorer retention than peptide-bound stationary phases, which denotes that binding is happening, thanks to the presence of the peptide on GB, discarding other possibilities like possible physisorption on glass or binding to free amino groups provided by the APTES layer. Based on all these findings, it was deduced that, for affinity-based washes, 40 °C could be a good temperature to keep retained MIN particles having highest affinity, while removing unreacted compounds.

Alternatively, ethanol was tested as mobile phase in order to assess MIN-peptide interactions in a non-aqueous medium. Hydrophobic binding should be minimised using ethanol as medium, and, therefore, another binding behaviour might be expected. As it can be deduced from Fig. 2b, the “U”-shaped graphs appreciable at Fig. 2a are not present in Fig. 2b. In the latter, stronger binding of MIN to all tested stationary phases can be observed at temperatures below 30 °C. In fact, maximum retention was observed at 4 °C rather than at 40 °C (Fig. 2b). When using ethanol, the hydrophilic/hydrophobic shift of MIN was not found to be determinant in the binding event, as opposed to water. It was also observed that low temperatures caused weak non-specific binding, which could be disrupted above 30 °C. Specific retention (interpreted as peak area ratios between control peptide/target peptide or blank column/target peptide) was maximised at 50 °C and 60 °C. Making a comparison between water and ethanol as mobile phases, it was observed that, at 50 °C, the control peptide/target peptide signal ratio was 5.37 using water and 1.93 using ethanol, whereas blank column/target peptide ratios were 3.96 and 1.79, respectively. It could be assumed that the higher the ratio, the more specific the interaction; therefore, it was found that water was providing better binding than ethanol.

All data shown in Fig. 2a were obtained using ultrapure water as mobile phase. However, in order to determine the influence of salt concentration of MIN-peptide binding, chromatographic experiments were performed using 25 mM PB and 0.1 M PBS at pH 7.4 as mobile phases. Results are shown in Fig. 2c and d. In both figures, higher binding is observed as temperature increases above 30 °C; however, a little difference can be appreciated when Fig. 2c and d are compared. At temperatures above the LCST MIN will turn hydrophobic, and, as a result, non-specific binding may be increased. This binding is stronger as salt concentration increases, probably due to the salting out effect, and that is why 25 mM PB buffer as mobile phase (Fig. 2c) provides higher peak areas than 0.1 M PBS (Fig. 2d), which shows almost full retention. Conversely, below 30 °C, MIN are more hydrophilic, and salt concentration contributes negatively to binding. Comparing results recorded using ultrapure water, 0.1 M PBS and 25 mM PB as mobile phases, the signal increase observed above 40 °C using water is not appreciable with the other mobile phases. Probably, the presence of phosphate salts or even NaCl are making hydrophobic interactions stronger, resulting in no binding difference between tested stationary phases (Fig. 2c and d).

It was also desirable to know which conditions could maximise MIN recovery at affinity-based washes after solid-phase synthesis. With this purpose, MIN-target peptide binding was examined at different mobile phases. Elution of MIN particles was measured at different pH and temperatures, obtaining the results illustrated in Fig. S1. MIN-peptide binding was maximum at pH below 5, whereas particle elution happened preferably at high pH. Moreover, like in previous experiments, decreasing the temperature favoured MIN elution. Based on this, a buffer having basic pH and low temperature could be recommendable to collect MIN particles after solid-phase synthesis.

### Characterisation of imprinted nanoparticles

It is well-known that hydrogels prepared with monomers such as NIPAm [33] or even TBAm [34] can undergo a reversible volume phase transition and they can swell in a solvent at low temperatures, whereas they shrink if the temperature is increased over the LCST. Below this temperature, polyNIPAm (LCST ≈ 32 °C) is water soluble [35], whereas above it, the polymer turns insoluble and it aggregates. Phase behaviour of the MIN was determined through turbidity experiments as detailed elsewhere [33]. As depicted in Fig. S2, absorbance changes with temperature confirm phase transition of MIN, thereby demonstrating the thermoresponsive character of the polymer. Based on presented results, LCST, defined at 10% absorbance [33], was found to be 33.4 ºC.

**Fig. 3:**
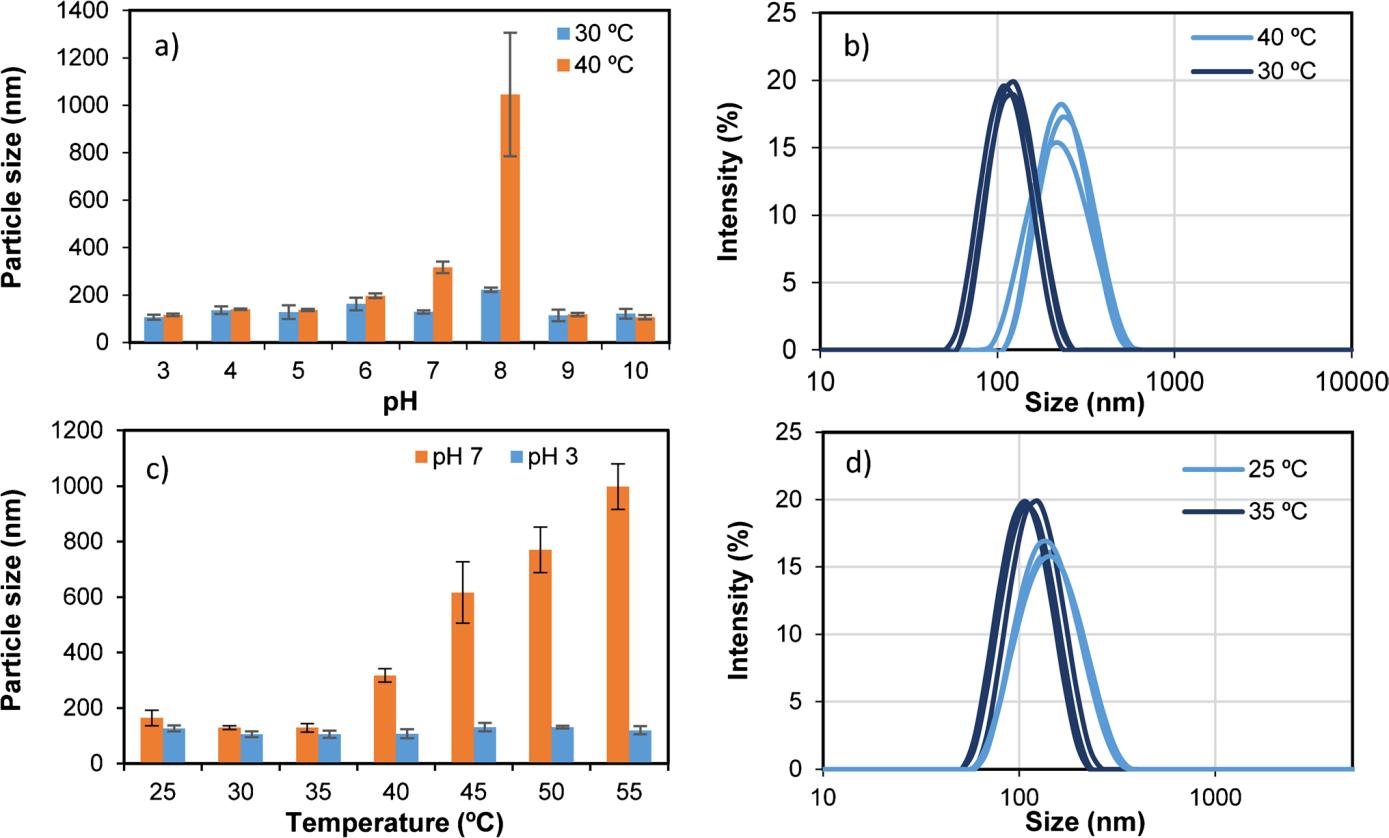
Particle size of imprinted nanoparticles measured **a** at pH values comprised between 3 and 10 at two different temperatures and **c** at temperatures comprised between 25 and 55 at two different pH. Particle size distribution data (*n* = 3) measured at **b** pH 7 (30 °C and 40 °C), and **d** pH 3 (25 °C and 35 °C)

Size analysis of imprinted nanoparticles was determined through dynamic light scattering analysis (DLS) using different pH and temperatures. To this end, MIN were suspended in 25 mM formate (at pH:3 and pH:4), 25 mM acetate (pH:5), 25 mM phosphate (pH:6, pH:7 and pH:8), and 25 mM carbonate buffers (pH:9 and pH:10). Particle size and polydispersity indexes were determined at all those pH, setting different temperatures, one of them below the LCST and the other one above. According to results presented in Fig. 3a, at most of tested pH values, no remarkable particle size difference was detected by DLS, except for pH 6, 7, and 8. At these values, the size was larger at 40 °C than at 30 °C, especially at pH 8. This may happen due to particle aggregation because of poor stability of the colloidal suspension. This aggregation is more evident at 40 °C, when particles have switched from a hydrophilic to a hydrophobic state. At this point, their water solubility decreases and, thereupon, they form aggregates. Figure 3b shows particle size difference using the same MIN suspension at two tested temperatures. Measurements performed at 40 °C were less reproducible due to particle aggregation, and relatively long sonication times were required between measurements to achieve good-quality signals. To determine how pH influenced particle aggregation, size analysis was performed at pH 3 and 7 at temperatures comprised between 25 and 55 °C (Fig. 3c). Due to the thermoresponsive nature of MIN, increasing temperature should give rise to smaller particles; however, the opposite happened above 40 °C for MIN suspended in PB (pH:7), which confirmed aggregation phenomena at neutral pH. Nevertheless, compared to pH 7, particle size was not so influenced by temperature at pH 3, which confirmed higher stability of MIN at this pH. Between 25 and 35 °C, no aggregation was perceived and, due to particle shrinking, a decrease in size from 164.0 ± 28.2 nm (PDI: 0.84 ± 0.02) to 129.0 ± 15.2 nm (PDI: 0.16 ± 0.03) was observed at pH 7 (Fig. 3d), while at pH 3, it decreased from 126.4 ± 10.5 (PDI: 0.79 ± 0.22) to 106.3 ± 15.2 nm (PDI: 0.32 ± 0.04).

**Fig. 4:**
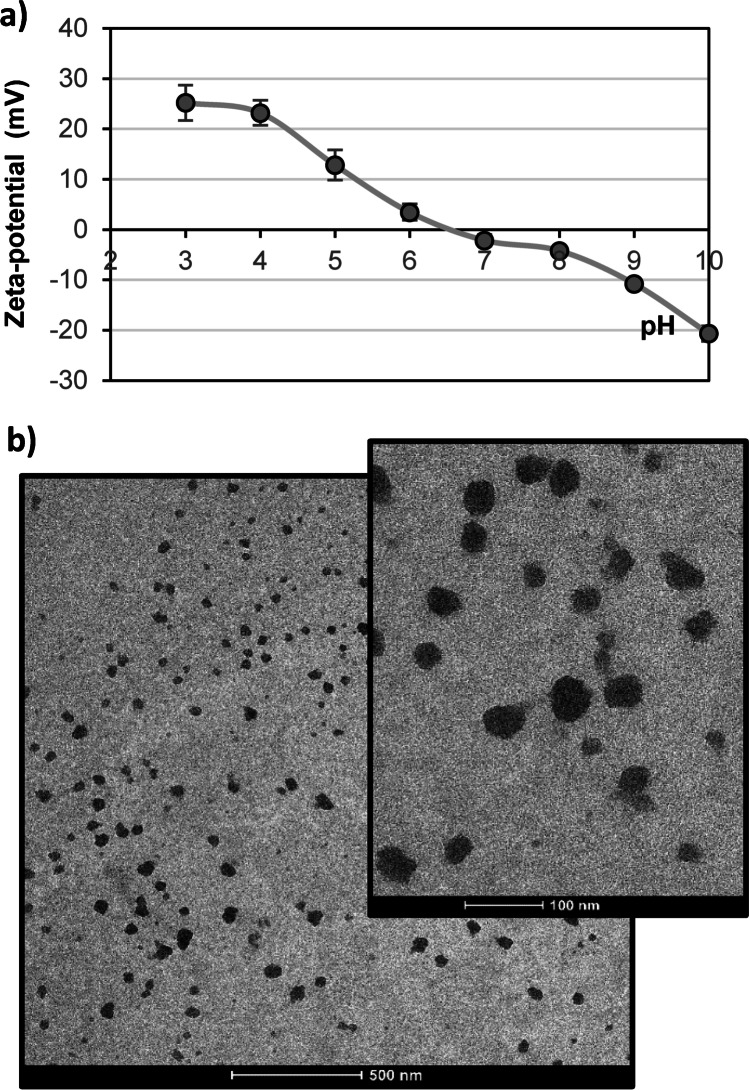
**a** Zeta-potential values recorded for a 0.1 mg mL^−1^ MIN suspension at different pH. **b** TEM images at different magnifications of developed imprinted nanoparticles

Particle aggregation may be attributed to weak repulsive forces between them, which may be related to a net effective surface charge close to neutrality. Normally, colloidal suspensions having a zeta-potential above 30 mV or below − 30 mV are considered moderately stable against aggregation because repulsive forces are high enough to prevent it [36]. Taking this into consideration, the zeta-potential of MIN suspensions was measured, which were prepared at different pH, as previously detailed for size analysis. As it can be observed in Fig. 4a, the isoelectric point of MIN is about 6.5, while the highest suspension stability will be reached at pH 3, where zeta-potential was found to be 25.22 ± 3.52 mV, or even at pH 10 (− 20.68 ± 1.55 mV). At pH 3, particle charge may be expected to be positive, since carboxylic groups coming from the monomer AA will be non-ionised, while amino groups of APMA will be positive. Instead, just the opposite may be expected at pH 10. TEM micrographs depicted in Fig. 4b show the spherical morphology of developed MIN nanoparticles having a size in the nanometre scale.

### Dot blot and Western blot experiments

**Fig. 5:**
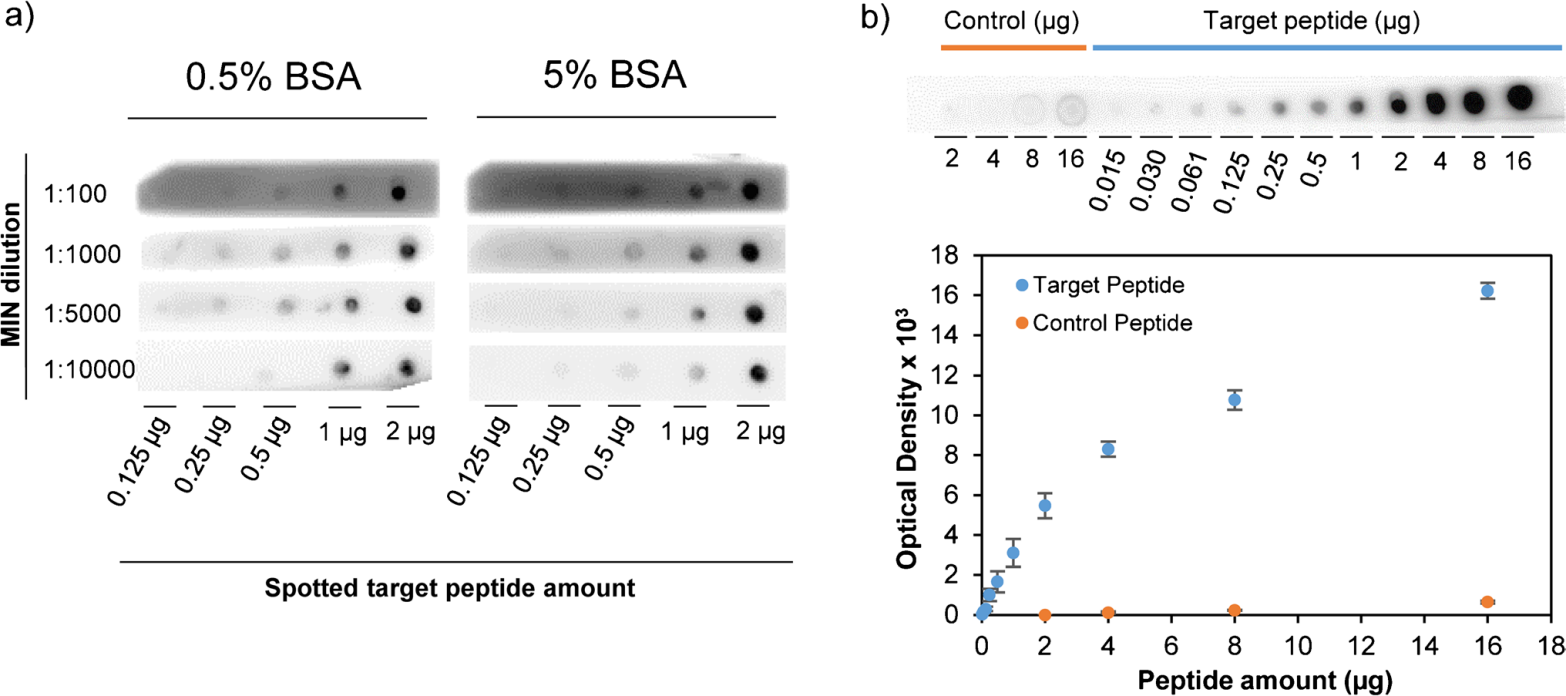
**a** Results obtained from dot blot experiments using different dilutions of MIN after membrane blocking with 0.5% or 5% BSA. **b** Dot blot experiment carried out using a single membrane spotted with detailed amounts (µg) of the target and control peptides. The graph below depicts the optical densities corresponding to each spot

In order to determine the best conditions for blotting assays, a series of dot blot experiments were carried out. Initially, the concentration of MIN that provided the highest signal with lowest background noise was determined. For this purpose, increasing amounts of the target peptide were spotted on a series of nitrocellulose membranes; then, they were blocked using 0.5% or 5% BSA; and, finally, they were incubated at RT in different dilutions of a stock solution of 510 mg L^−1^ of MIN. As it can be observed in Fig. 5a, a 1:100 dilution was not enough to properly differentiate signal from the background, while diluting MIN 1:10,000 provided too low sensitivity. Thus, dilutions that ranged from 1:1000 to 1:5000 may be suitable. In addition, it was observed that results were quite similar regardless the percentage of BSA, besides 5% BSA provided higher background noise than 0.5%. Further experiments were also carried out reducing even more than 0.5% the BSA level; however, the signal to noise ratio did not improve compared to 0.5%, thereby establishing this value as pertinent. Finally, temperature effect on MIN binding was also assessed. Figure S3 illustrates optical densities measured for each spotted amount of peptide after incubating the membranes in MIN suspensions at different temperatures. As shown in the figure, incubation at 35 °C provides the highest sensitivity, which matches with the thermoresponsive character of developed MIN.

At optimised conditions, another dot blot experiment was run using membranes having blots with different amounts of the target peptide and the peptide used as control (Fig. 5b). Chemiluminescence signal was dependant on the spotted amount of peptide on the membrane obtaining a saturation like behaviour. For comparison, the control peptide was also spotted in the same membrane; however, the obtained signal was negligible in comparison with the target.

**Fig. 6:**
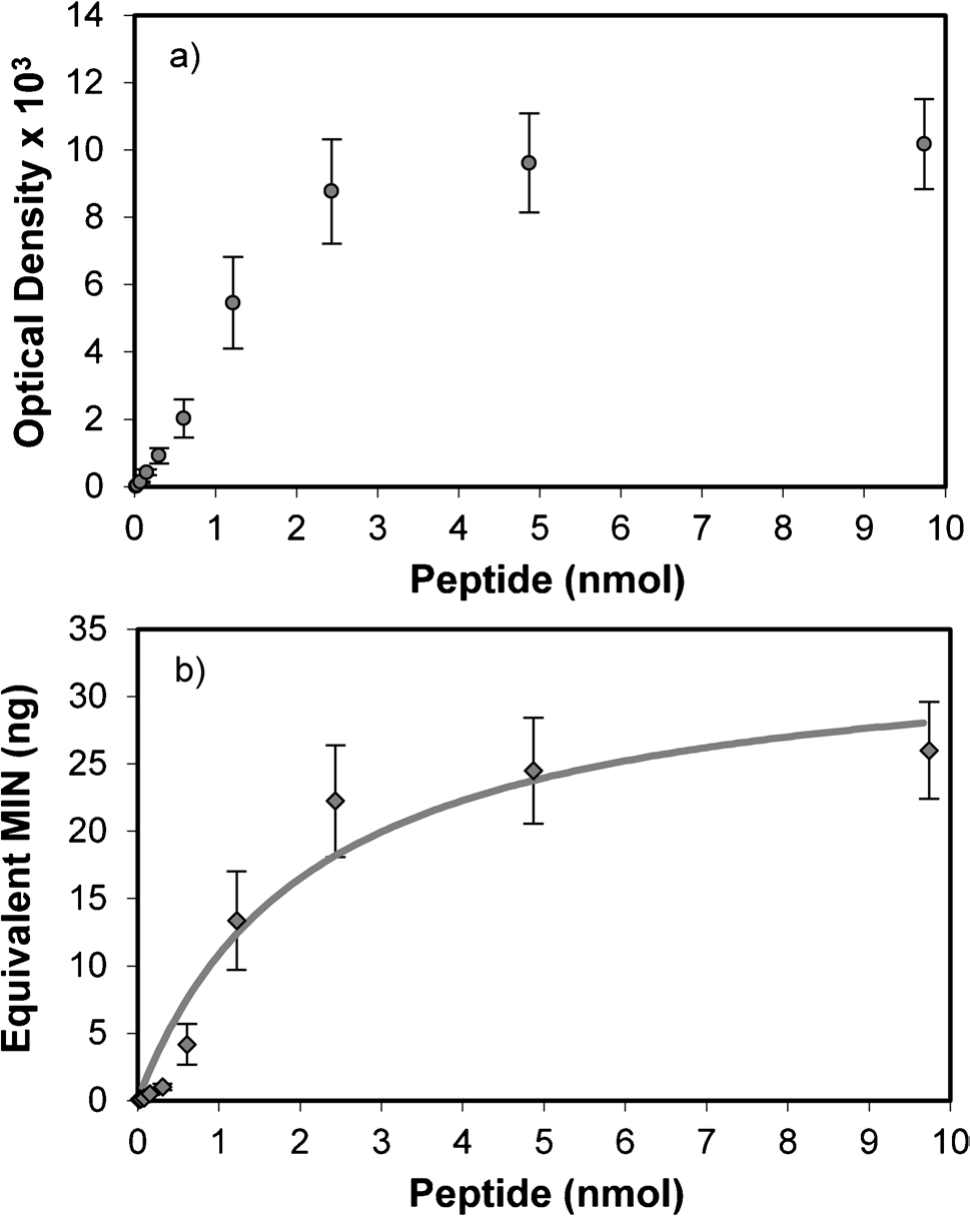
**a** Optical densities recorded as a function of the spotted amount of peptide after membrane incubation in a 0.1 µg mL^−1^ suspension of MIN at 35 °C for 24 h. **b** Equivalent amount of MIN (ng) bound to each peptide spot having the illustrated nmoles

The dot blot assay was also used as a tool to assess affinity of developed MIN. To this end, it was necessary to first determine the chemiluminescent specific activity of MIN, which may be expressed as optical density (OD) increment as a function of MIN amount on the membrane. This was carried out spotting increasing amounts of biotinylated nanoparticles (0.1 ng to 50 ng) on nitrocellulose membranes and, then, allowing them to dry at 40 °C for proper fixation. Finally, membranes were left to react with the avidin–biotin/HRP complex and chemiluminescence signal was acquired as detailed in the “Experimental” section. Signal dependence to the amount of MIN on the membrane showed two linear regions with different slopes. The first one, between 0.1 and 1 ng of MIN, presented a slope or specific activity of 952 OD ng^−1^, whereas the second, between 1 and 25 ng, revealed a specific activity of 373 OD ng^−1^. After knowing this, another dot blot assay was performed spotting increasing peptide amounts in a nitrocellulose membrane and incubating them in a 0.1 µg mL^−1^ solution of MIN. Figure 6a depicts obtained OD values versus the peptide amount fixed on the membranes; instead, Fig. 6b shows the equivalent amount of MIN bound to each peptide spot, which was calculated using mentioned specific activity data. The experimental data depicted in Fig. 6b was adjusted to a one-site binding hyperbola using the GraphPad 9.0 software using the following expression:

$$B=\frac{{B}_{max}F}{{K}_{D}+F}$$

where B and F represent bound and free concentrations of a ligand, Bmax is the maximum specific binding, and K_D_ is the equilibrium dissociation constant. This model has been adapted to the particular situation of the present work, where binding experiments have been conducted on a nitrocellulose membrane rather than in solution. Fitting parameters revealed an apparent Bmax value of 34.29 ± 4.14 ng of equivalent MIN, which may give an idea about the maximum amount of MIN capable of being bound to the peptide. Making the assumption that 34.29 ng of equivalent MIN will bind to the maximum peptide amount used in the assay, i.e. 10 nmol of peptide, the estimated number of sites could be expressed as 0.29 nmol of peptide per ng of equivalent MIN. K_D_ value obtained from data fitting was found to be 2.15 ± 0.70 nmol of peptide. This should not be understood as a real dissociation constant, but rather a way to make an approximation to material affinity for comparative purposes.

**Fig. 7:**
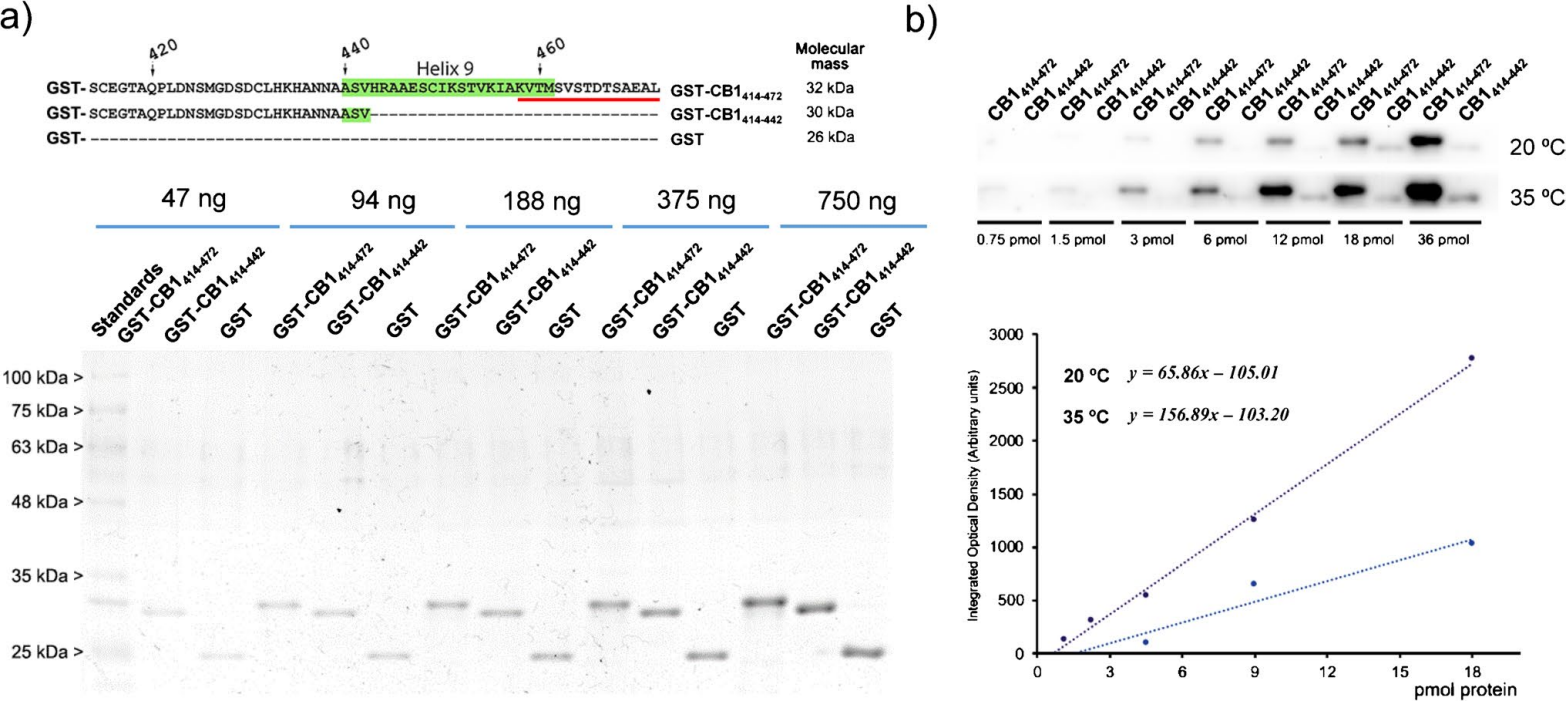
**a** Purity and molecular weight analysis of recombinant GST-CB1_414-472_ and GST-CB1_414-442_ fusion proteins using SDS–polyacrylamide gels. **b** Results of a Western blot assay of developed anti-CB1 artificial antibodies against increasing amounts of fusion proteins. The graph below represents the corresponding optical densities for each level of the target protein

In order to better assess the specificity and sensitivity of developed anti-CB1 MIN generated against the carboxy-terminus of the CB1 receptor (458-KVTMSVSTDTSAEAL-472), the fusion protein GST-CB1_414-472_ was used as target for Western blot assays. To this end, the sequence of amino acids 414 to 472 was fused to the protein GST (glutathione S-transferase). As negative control, the fusion protein GST-CB1_414-442_ was employed, which did not have the C-terminus sequence (458–472) of the CB1 receptor. Figure 7a shows the sequences of fabricated fusion proteins as well as the results obtained from their purity analysis. It can be corroborated that molecular masses obtained empirically were the expected ones for produced proteins. Figure 7b depicts representative data relative to a Western blot assay using developed MIN. An intense chemiluminescence signal can be observed in Fig. 7b which shows linear dependence to the concentration of GST-CB1_414-472_ loaded per lane. Conversely, lanes loaded with GST-CB1_414-442_ presented almost negligible signals, since this protein did not carry the target epitope for being devoid of last 30 amino acids at the C-terminus. The signal (optical density) provided by MIN was linear between 0.75 and 18 pmol at both tested temperatures; however, sensitivity was remarkably higher at 35 °C. The detection limit (explained as the cut-off point of the curves with the abscissa axis) was found to be 1.6 and 0.67 pmol at 20 °C and 35 °C, respectively. Based on these findings, we can confirm that solid-phase imprinting of peptides has proven useful to develop MINs that behave as natural antibodies against the C-terminus of the CB1 receptor. Nevertheless, efforts are still necessary to improve this recent methodology to produce high-affinity MIN with higher yields. Developed MIN nanoparticles have been validated in assay analogues to which commonly are used for natural antibodies. Relying on the good results provided by this material, it may be further exploited in the near future for specific localisation and quantification of this receptor through bioimaging techniques in cells and tissues.

## Conclusions

Artificial antibodies against the membrane receptor CB1 have been developed in this work, having an epitope that binds selectively to the C-terminus extreme. So far, MIN-based artificial antibodies have mainly focused on globular proteins; instead, the solid-phase imprinting of epitopes has exploited here as a proof of concept to develop MIN against linear sequences of membrane proteins. The developed imprinted material has proven efficient for binding the target protein through the intracellular region of the receptor (458-KVTMSVSTDTSAEAL-472) in Western blot experiments. Control GST fusion proteins not having this sequence were unable to bind the plastic antibody, thereby demonstrating that the epitope-imprinting approach was a good choice to accomplish the pursued goals. The methodology followed in this work has served to fabricate high-quality antibodies that will be very likely applied in other bioanalytical techniques. Potential benefits presented by the produced material could be exploited for receptor isolation and purification from soluble extracts of this protein, working like antibodies in immunoprecipitation approaches. Additionally, they could even serve as recognition elements for protein separation in affinity chromatography, and they could also be appropriate for bioimaging. All this will definitely help in obtaining valuable information concerning receptor localisation and distribution in biological tissues.

## Supplementary Information

Below is the link to the electronic supplementary material.

Supplementary file1 (DOCX 160 KB)

## Data Availability

Data sharing not applicable to this article as no datasets were generated or analysed during the current study.
