# Supplementary material for: Solid-phase synthesis of imprinted nanoparticles as artificial antibodies against the C-terminus of the cannabinoid CB1 receptor: exploring a viable alternative for bioanalysis
Source: Mikrochim Acta. 2021 Oct 7;188(11):368. doi: 10.1007/s00604-021-05029-z (PMC8497319; doi:10.1007/s00604-021-05029-z)
Supplement: Supplementary file 1 — Supplementary file1 (DOCX 160 KB) [file 604_2021_5029_MOESM1_ESM.docx]

**Electronic Supplementary Material**

**Solid-phase synthesis of imprinted nanoparticles as artificial antibodies against the C-terminus of the cannabinoid CB1 receptor: exploring a viable alternative for bioanalysis**

Alberto Gómez-Caballero^1,^*, Ainhoa Elejaga-Jimeno^1^, Gontzal García del Caño^2^, Nora Unceta^1^, Antonio Guerreiro^3^, Miquel Saumell-Esnaola^4^, Joan Sallés^4,5^, M. Aránzazu Goicolea^1^ and Ramón J. Barrio^1^.

^1^ Department of Analytical Chemistry, University of the Basque Country UPV/EHU, 01006 Vitoria-Gasteiz (Álava), Spain.

^2^ Department of Neurosciences, Faculty of Pharmacy, University of the Basque Country UPV/EHU, 01006 Vitoria-Gasteiz (Álava), Spain.

^3^ MIP Diagnostics, Leicester, UK.

^4^ Department of Pharmacology, Faculty of Pharmacy, University of the Basque Country UPV/EHU, 01006 Vitoria-Gasteiz (Álava), Spain.

^5^ Centro de Investigación Biomédica en Red de Salud Mental (CIBERSAM), 28029 Madrid, Spain.

*Corresponding author. Tel: +34-945013857

*E-mail address:* a.gomez@ehu.eus

**Experimental**

*Apparatus*

Polymers were synthesised using UV lamps model Summer Glow HB175 (75 W) from Hapro (The Netherlands). The instrument Zetasizer Ultra acquired from Malvern Panalytical (France) was used for size analysis by dynamic light scattering (DLS) and zeta potential measurements. Transmission electron microscopy (TEM) images were acquired using a TECNAI G2 20 TWIN microscope operating at 200 kV and equipped with LaB_6_ filament purchased from FEI (USA). For this purpose, a drop of MIN suspension was spread onto a TEM copper grid (300 Mesh) coated with a pure carbon film and dried at room temperature.

UV‑Vis measurements were performed using a Cary 60 UV-Vis spectrophotometer equipped with a 1 m long fibre optic probe and a 10 mm path length stainless steel probe tip. For lower critical solution temperature analysis, the spectrophotometer was connected to a water-thermostatted cell holder connected to a circulation bath with Peltier-driven temperature control.

HPLC experiments were carried out using an Agilent 1200 series HPLC system comprised of an Agilent 1260 infinity binary pump, vacuum degasser, a Rheodyne manual sample injector with a loop of 20 μL, and a fluorescence detector (FD). The software Agilent LC ChemStation (Agilent Technologies, USA) was used for system control and data analysis. MS-QTOF measurements were acquired using an Agilent 6530 hybrid quadrupole-time of flight mass spectrometer with an ESI (electrospray ionisation) Agilent Jet Stream source (Agilent Technologies, Waldbronn, Germany).

*Fabrication of fluorescent MIN*

For HPLC experiments, in order to provide synthesised nanoparticles with fluorescence, the monomer N-fluoresceinyl acrylamide was used, which was synthesised as detailed by [2]. For this purpose, fluoresceinamine and acryloyl chloride were purchased from Merck (Spain). High-resolution mass spectrum (ESI-QTOF-MS) for the obtained product provided the following *m/z* data: [M + H]^+^ 402.0982, calculated mass: 401.0899, found mass: 401.0911, difference: 3.01 ppm.

Synthesised nanoparticles were derivatised with N-fluoresceinyl acrylamide instead of MIN biotinylation (as described in section: Solid-phase synthesis of imprinted nanoparticles). For that, after the warm washes to remove unreacted compounds, the MIN-containing GB were added to a round bottom glass tube containing a 0.1 mg mL^-1^ solution of N-fluoresceinyl acrylamide in 25 mL of PB. Next, the mixture was purged with N_2_ for 30 min and it was irradiated for 15 min using two UV lamps as previously detailed for nanoparticle synthesis. This step allowed for grafting the fluorescent monomer on the surface of MIN. Then, fluorescent MIN were collected and concentrated as described before, obtaining a final 10 mL suspension of MIN in formate buffer.

*Turbidity measurements*

For turbidity measurements, absorbance of a 0.25 mg mL^-1^ MIN suspension at 600 nm was measured as a function of temperature in the range comprised between 10-60 ºC. To this end, the water-thermostatted cell holder was set to the desired temperature and absorbance was monitored until signal stabilisation.

*Production and purification of GST-CB1_414-472_ and GST-CB1_414-442_ fusion proteins*

Following the supplier's instructions, pGEX-P1 plasmids carrying the GST-CB1_414-472_ and GST-CB1_414-442_ protein-coding sequences were heat-shocked into the bacterial strain *Rosetta(DE3)pLysS* (Novagen, Spain) derived from strain BL21. After that, they were seeded on Lysogeny Broth‑Agar plates (LB-Agar) containing 100 µg mL^-1^ ampicillin and 34 µg mL^-1^ chloramphenicol (Merck, Spain), and they were allowed to grow for 24 hours at 37 °C. The double selective pressure of antibiotics can happen thanks to the presence of ampicillin resistance in the plasmid pGEX-6P1, and chloramphenicol resistance in the plasmid pRARE, furthermore, the latter contains tRNA coding sequences for eukaryotic codons rarely found in *E. coli*. From the LB-Agar culture, a clone was isolated and suspended in 5 mL of LB containing 100 µg mL^-1^ ampicillin and 34 µg mL^-1^ chloramphenicol, in order to prepare a pre-culture of *Rosetta(DE3)pLysS* bacteria. After overnight growth at 37°C, 1 mL of this pre-culture was inoculated into 50 mL of LB containing ampicillin and chloramphenicol. The culture was grown at 37°C and optical density at 600 nm (OD600) was monitored every 30 min by UV-Vis spectroscopy until a value of 0.5-0.6 was reached. At this point, isopropyl-β-D-thiogalactoside (Merck, Spain) was added to the culture to reach a final concentration of 0.1 mM, as well as an additional dose of ampicillin. When the OD600 reached a value of 1.2, a pellet was obtained by centrifugation at 3500 x g for 20 min, discarding the supernatant.

Bacterial pellets were subjected to 3 freeze/thaw cycles, immersing the pellet containing vial in isopentane at -80ºC for freezing and in ice for thawing. Then, the pellet was resuspended in 3 mL lysis buffer, which consisted of 2.5 mM EDTA, 1 mL of protease inhibitor cocktail (Merck, Spain) and about 1 mg pefabloc (Merck, Spain) prepared in 20 mL of 50 mM tris-buffered saline (TBS) at pH 7.6. The mixture was vortexed, next another 4 mL of lysis buffer were added, and then vortexed again vigorously. Thereafter, three sonication cycles of 20s were applied at 80V while the tube was kept in ice, and Triton X-100 (Merck, Spain) was added until reaching 1% (v/v). The mixture was, then, incubated at 4ºC for 30 min on a swing shaker. Afterwards, the protein extract was centrifuged at 4ºC and 30,000 x g for 30 min, and the supernatant (protein extract) was collected for purification.

GST-CB1_414-472_ and GST-CB1_414-442_ fusion proteins were purified using glutathione-coated magnetic beads (Thermo Fisher, Spain), strictly following the procedure recommended by the supplier. Finally, GST fusion proteins were eluted with 4 mL of 50 mM reduced glutathione (Merck, Spain) in elution buffer (125 mM Tris-HCl, 150 mM NaCl, 1 mM DTT, 1 mM EDTA, pH 7.4) per lysate obtained from 50 mL of bacterial culture. The amount of protein was determined using the Bradfford micromethod, using Protein Assay Dye Reagent Concentrate (Bio-Rad, Spain) and γ-globulin (Bio-Rad, Spain) as standard.

*Purity analysis of recombinant proteins*

The purity and molecular weight of GST-CB1_414-472_ and GST-CB1_414-442_ fusion proteins were analysed by electrophoresis on SDS-polyacrylamide gels. For this purpose, one volume of the purified recombinant protein GST-CB1_414-472_, GST-CB1_414-442_ or GST was mixed with three volumes of urea 4x buffer, containing 300 mg urea, 50 mg sodium dodecyl sulfate (SDS), 120 mg dithiothreitol (DTT), 500 µL Tris-HCl 100 mM at pH:8.0, 6 µL of 1 % (w/v) bromophenol blue and 4.5 mL of ultrapure water. Then, protein concentration of each solution was adjusted to 75 ng µL^-1^ using urea 1x buffer (urea 4x diluted 1:4) containing 12.5% glycerol. After heating the proteins at 60°C for 5 minutes, 4 consecutive 50 % (v/v) dilutions were made using 1x urea buffer. Then 10 µL of each dilution were loaded per lane onto 12% SDS-polyacrylamide gels and the proteins were resolved by electrophoresis at 120 V on a Protean II xi (Bio-Rad, Spain). Following electrophoresis, the gels were stained with Coomassie blue (Bio-Rad, Spain) according to the manufacturer's instructions. The obtained results revealed the presence of a single net band that corresponded to each of the three tested proteins, whose migration was consistent with their theoretical molecular mass (Fig.7a).

**Fig. S1.** Registered peak areas for a 0.25 mg mL^-1^ MIN suspension using peptide-bound GB as stationary phase. 100% aqueous buffers at depicted pH were used as mobile phase. Measurements were conducted at 20 ºC and 40 ºC.

a)


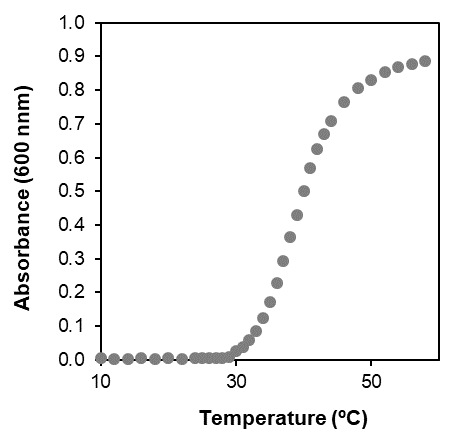

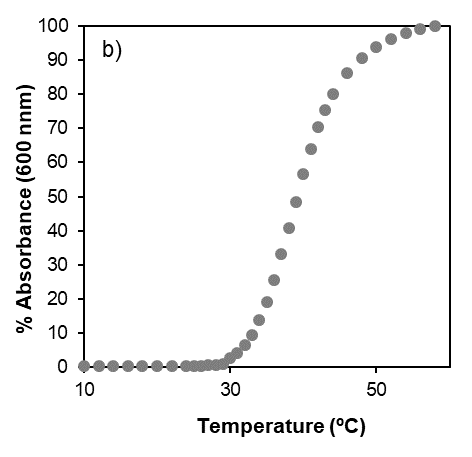


**Fig. S2**. Registered a) absorbance and b) normalised absorbance data for a 0.25 mg mL^-1^ MIN suspension in 0.1 M PBS at temperatures ranging from 10 ºC to 60 ºC. Shown data correspond to stable absorbance values registered after setting the temperature to the desired value and signal monitorisation until stabilisation.


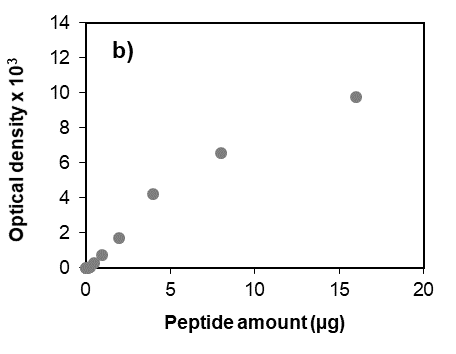

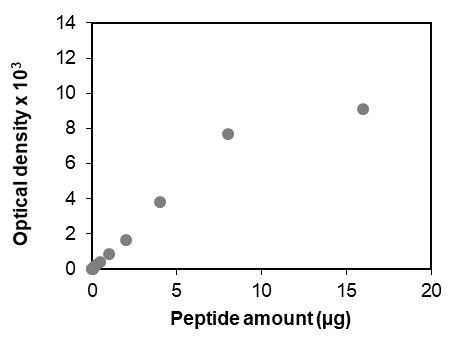

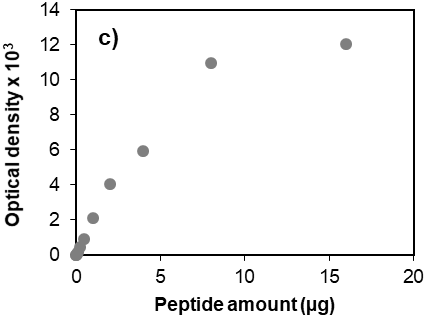

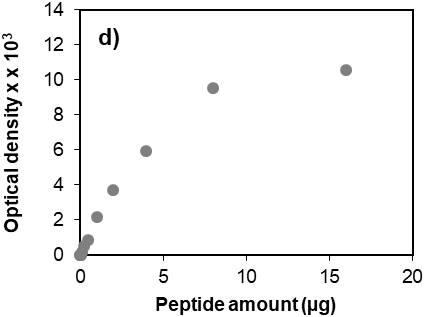


**a)**

**Fig. S3.** Measured optical densities after dot blot assays carried out incubating nitrocellulose membranes with a MIN suspension in 0.1 M PBS at a) room temperature, b) 4 ºC, c) 35 ºC and d) 45 ºC. Membranes were spotted with the shown amounts of the target peptide.

**References**

1. Xu J, Haupt K, Tse Sum Bui B (2017) Core-Shell Molecularly Imprinted Polymer Nanoparticles as Synthetic Antibodies in a Sandwich Fluoroimmunoassay for Trypsin Determination in Human Serum. ACS Appl Mater Interfaces 9:24476–24483. https://doi.org/10.1021/acsami.7b05844

2. Canfarotta F (2016) Molecularly Imprinted Nanoparticles for Diagnostic Applications. Dissertation, University of Leicester
